# Emergent Toxins in North Atlantic Temperate Waters: A Challenge for Monitoring Programs and Legislation

**DOI:** 10.3390/toxins7030859

**Published:** 2015-03-16

**Authors:** Marisa Silva, Vijaya K. Pratheepa, Luis M. Botana, Vitor Vasconcelos

**Affiliations:** 1 CIIMAR/CIMAR—Interdisciplinary Center of Marine and Environmental Research, University of Porto, Rua dos Bragas 289, Porto 4050-123, Portugal; E-Mails: marisasilva17@gmail.com (M.S.); vpratheepa@gmail.com (V.K.P.); 2 Faculty of Sciences, University of Porto, Rua do Campo Alegre, Porto 4169-007, Portugal; 3 Department of Pharmacology, Faculty of Veterinary, University of Santiago of Compostela, Lugo 27002, Spain; E-Mail: luis.botana@usc.es

**Keywords:** emergent toxins, monitoring, new vectors, legislation

## Abstract

Harmful Algal Blooms (HAB) are complex to manage due to their intermittent nature and their severe impact on the economy and human health. The conditions which promote HAB have not yet been fully explained, though climate change and anthropogenic intervention are pointed as significant factors. The rise of water temperature, the opening of new sea canals and the introduction of ship ballast waters all contribute to the dispersion and establishment of toxin-producing invasive species that promote the settling of emergent toxins in the food-chain. Tetrodotoxin, ciguatoxin, palytoxin and cyclic imines are commonly reported in warm waters but have also caused poisoning incidents in temperate zones. There is evidence that monitoring for these toxins exclusively in bivalves is simplistic and underestimates the risk to public health, since new vectors have been reported for these toxins and as well for regulated toxins such as PSTs and DSTs. In order to avoid public health impacts, there is a need for adequate monitoring programs, a need for establishing appropriate legislation, and a need for optimizing effective methods of analysis. In this review, we will compile evidence concerning emergent marine toxins and provide data that may indicate the need to restructure the current monitoring programs of HAB.

## 1. Introduction

### 1.1. Harmful Algal Blooms: General Description

Phytoplankton may develop blooms in marine coastal waters with seasonal, regional and species-specific features [1]. Several factors, which are not yet entirely understood, promote these blooms, but in recent decades these occurrences have tended to be more frequent, persistent and intense [2,3,4,5,6]. Climate change, eutrophication and cysts, together with alien species transported in ballast waters, are noted as important contributors [7]. Blooms can be classified as benign or harmful according to their impact on the ecosystem, on public health and on the economy. Benign algal blooms lead to an increase of primary producers boosting the richness of the ecosystem, whereas Harmful Algal Blooms (HAB) have adverse consequences [8,9]. So far, about 5000 species of phytoplankton have been distinguished, 300 of which form blooms, and are reported as toxic, noxious or as being a nuisance [1,10,11,12].

A phytoplankton bloom is a complex community that can be monospecific or composed of several different species [13]. In both cases, harmful species may or may not be present. Toxic blooms produce secondary metabolites that may help them outcompete similar species or have deleterious effects on predators [14,15,16,17]. These toxins can accumulate in the food-chain and cause poisoning incidents to humans through harvested shellfish or other seafood present in the bloom area [18,19]. HABs affect the fishing and aquaculture industries by causing high mortalities in fish and invertebrates through mechanical damage due to their spiny conformation or mucilage production, and by causing lesions or obstruction of the gills [20,21]. As an example, the diatoms *Chaetoceros concavicornis* and *C. convolutus*, can cause fish mortalities at the very low concentration of 5 cells/mL [22].

A bloom can also create anoxic zones when it is very extensive and enters into senescence, thereby causing mortalities or deviation of fish migration routes [23,24]. An example of this is in the Gulf of Mexico, where the Mississippi River delivers heavy loads of urban and agricultural runoff leading to an increase in nitrogen and phosphorus levels and fueling phytoplankton growth. This influx causes extensive blooms whose decomposition eliminates oxygen faster than it can be replaced thereby forming dead zones [24].

HABs may cause huge economic losses in the tourism sector even when blooms are not a risk for humans or other organisms by producing foams, mucilage, repellent odors or altering the water color [25,26,27,28,29,30]. They can also affect an entire ecosystem by creating regions of anoxia, causing death by mechanical block preventing micro invertebrates to feed, affecting the reproduction of predators, benthic anoxia, sea grass die-off, and the alteration of food web function [23,31,32,33,34].

Regarding public health, a need for guidelines led to the establishment of international regulations resulting in mandatory and frequent monitoring of the most common syndromes: Paralytic Shellfish Poisoning (PSP), Amnesic Shellfish Poisoning (ASP) and Diarrheic Shellfish Poisoning (DSP) [35,36,37]. Nowadays, owing to these regulations, the cases of human intoxications are sporadic and are mostly due to illegal harvest and/or confusion of toxic species with non-toxic ones, *i.e.*, failure of harvest and consumption prohibitions implemented by national health authorities [38,39].

The establishment of guideline values for marine toxins follows some procedures that take into account the toxicity values produced through laboratory assays, data on incidence, prevalence, seasonal variation and vectors of the toxin obtained through field work. The monitoring of biotoxins is usually evaluated through phytoplankton counting and the testing of bioaccumulation of toxins in bivalves. However, there can be a lack of important data since some toxins are produced by bacteria (Tetrodotoxin (TTX)) and others are produced by benthic dinoflagellates (Ciguatoxin (CTX), Palitoxin (PTX)). Moreover, vector species that are not normally monitored, such as gastropods, crustaceans and fish, should be included in risk assessments since other toxin uptake routes, apart from filter feeding, are present in marine ecosystems [40,41]. This risk analysis is the key for the proposal of new guideline values and this procedure has to be done in accordance with international guidelines and institutions, such as the European Union Reference Laboratory for Marine Biotoxins (EURLMB) and the European Food Safety Authority (EFSA), coordinated by United Nations Organizations, such as the Food and Agricultural Organization of the United Nations (FAO), Intergovernmental Oceanographic Commission of UNESCO (IOC) and the World Health Organization (WHO). The EURLMB coordinates the activities of a network of the National Reference Laboratories (NRL) which is established in each EU Member State, regarding the methodologies applied to control marine biotoxins in shellfish in order to protect public health and guarantee a maximum level of food safety. The EFSA is the keystone of European Union (EU) risk assessment regarding food and feed safety. In close collaboration with national authorities and in open consultation with its stakeholders, EFSA provides independent scientific advice and clear communication on existing and emerging risks.

In this review we explore the challenges of HABs, more specifically the problem of the emergent toxins, as evidence for their presence in temperate waters has become more substantive resulting in the need for new monitoring programs and the development of more sensitive and rapid analysis methods associated with a revised legislation in order to avoid social and economic consequences.

### 1.2. Emergent Toxins

There is urgency in the study of emergent toxins as the rise of water temperature, together with anthropogenic impacts, may allow for the dispersion and the establishment of new populations of highly toxic organisms [3,6,42,43,44]. These phycotoxins have many routes of uptake in humans, the most common one being via ingestion. The majority of these toxins are heat-stable, whereby cooking processes do not affect their structure or function. Dermal and respiratory exposure also has to be considered as some biotoxins can form aerosols. This is the case with PTX, which causes the development and aggravation of lung diseases affecting mainly coastal and fishermen populations [45,46,47,48]. In the following paragraphs, we will describe the chemical structure of TTX, PTX, CTX and Ciclic Imines (CI), action modes and symptoms in humans.

#### 1.2.1. Tetrodotoxin

**Figure 1:**
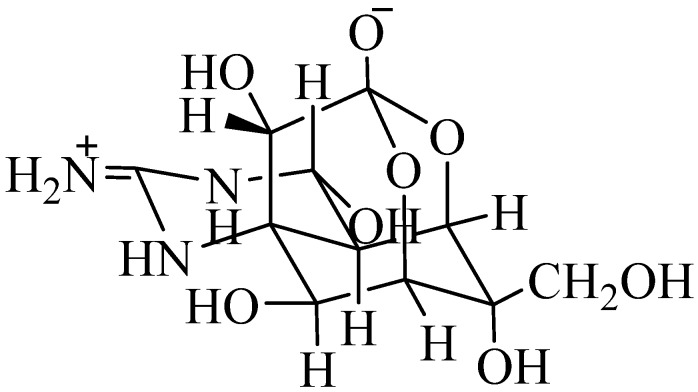
Tetrodotoxin (TTX) structure modified from Noguchi 2008 [55].

TTX is a non-proteinaceous neurotoxin with a molecular weight of 319.3 Da. TTX was first isolated in 1950 by Yokoo as a crystalline prism, from the toxic puffer fish and named after the puffer fish family Tetraodontidae [49,50]. The structure of TTX (Figure 1), was identified after the independent findings by several researchers, namely Goto *et al.* (1965), Tsuda *et al.* (1964) and Woodward (1964) [51,52,53]. TTX is a colorless, crystalline-weak basic substance with a molecular formula of C_11_H_17_O_8_N_3_ and has 30 analogues or derivatives which have been separated from puffer fish, newts, frogs and other TTX bearing organisms [54]. It is found in phylogenetically different marine and terrestrial organisms from six different phyla [55]. The widespread occurrence indicates that the origin of TTX may be exogenous [56,57,58]. The structure of TTX is characterized by a positively charged guanidinium group and a pyrimidine ring that may help TTX to work as a specific blocker of voltage gated sodium channels. Intoxication of TTX occurs within hours and may progress from localized numbness at the mouth shortly after ingestion to vomiting, strong headache, muscle weakness, respiratory failure, hypotension and even death [59]. As there is no antidote available, the main objective is to keep the patient alive in the first 24 h after intoxication of TTX occurs, with ventilator and hemodynamic support, as well as the correction of any possible cardiac arrhythmias, resulting in the mandatory stay in an intensive care unit [59,60,61].

#### 1.2.2. Palytoxin

**Figure 2:**
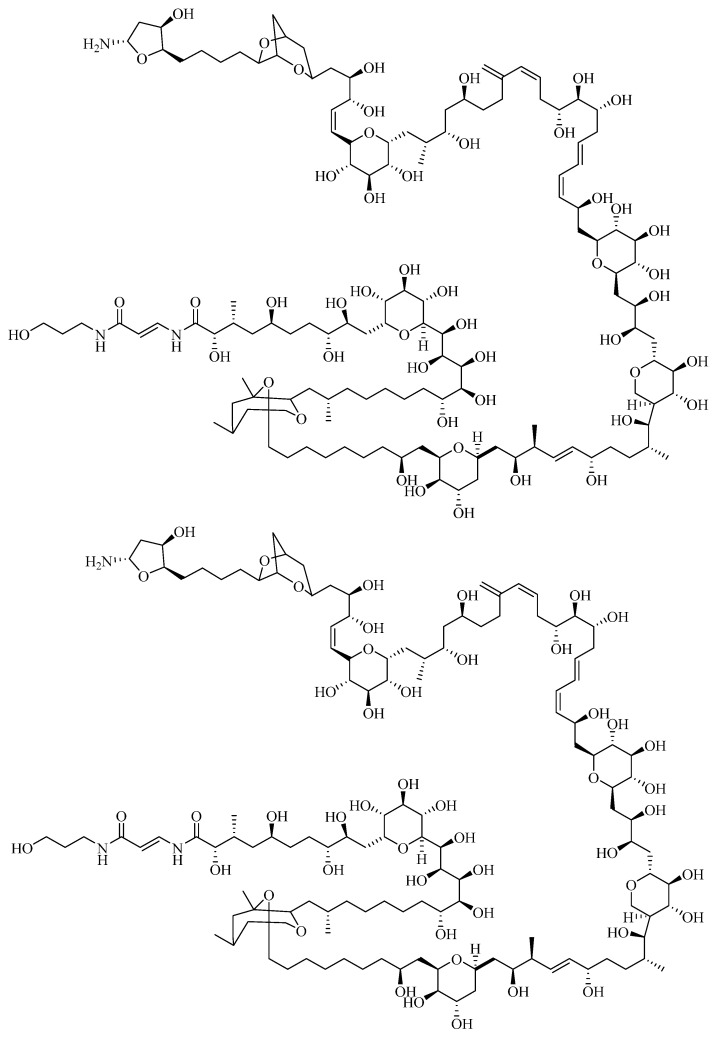
Palytoxin structure modified from Ramos and Vasconcelos 2010 [66].

PTX is a non-proteinaceous marine toxin which is mainly produced by marine zoanthids (soft corals) of the genus *Palythoa* [62]*.* Initially they were found only in Hawaii and Japan but the occurrence of PTX and its analogues is reported worldwide [63,64,65]. PTX is also produced by dinoflagellates (*Ostreopsis* spp.) and found in other organisms, such as fish [66,67]. Its structure was first described in 1981 [63,68]. PTX has a polyketide structure (Figure 2) with both lipophilic and hydrophilic moieties. The general chemical formula of PTX is C_129_H_233_N_3_O_54_ consisting in a long, partially unsaturated aliphatic backbone, containing cyclic ethers, 64 chiral centers, 40-42 hydroxyl and 2 amide groups. Many different analogues of PTX, such as isobaric PTX, ostreocin-D, ovatoxin (a to f), mascarenotoxins, ostreotoxin-1 and 2, homopalytoxin, bishomopalytoxin, neopalytoxin, deopalytoxin and 42-hydroxypalytoxin are known and the molecular weights vary depending on the species from which they are produced, ranging from 2659 to 2680 Da [69,70,71,72]. PTX has ultraviolet absorption at a wavelength of 233 and 263 nm and is heat-stable [69,73]. Palytoxin causes intoxication called clupeotoxism due to the consumption of clupeoid fish, such as sardines, herrings and anchovies [74]. Symptoms of PTX-group toxins include vasoconstriction, hemorrhage, myalgia, ataxia, muscle weakness, ventricular fibrillation, ischemia and death [75,76]. Moreover, Rhabdomyolysis syndrome is pointed out as being the most commonly reported complication after a poisoning incident with PTX [77]. This life threatening condition consists of a loss of intracellular contents into the blood plasma, causing injury to the skeletal muscle, with the worst cases resulting in renal failure and disseminated cardiovascular coagulation. Staying well-hydrated is strongly advised for the prevention of this condition [78].

#### 1.2.3. Ciguatoxin

**Figure 3:**
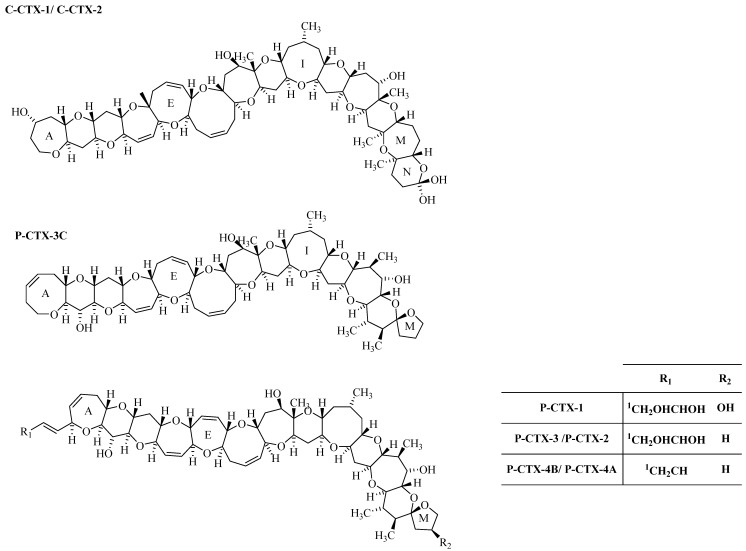
Structures Caribbean (C) and Pacific (P) CTX-group toxin. The energetically less favored epimers, P-CTX-2 (52-epi P-CTX-3), P-CTX-4A (52-epi P-CTX-4B) and C-CTX-2 (56-epi C-CTX-1) are indicated in parenthesis. Modified image from Lewis, 2001 [81]. Copyright 2001, Elsevier.

CTXs are reef toxins produced by the dinoflagellate *Gambierdiscus* spp. in warm, tropical or subtropical waters [79]. A three letter code with prefix is used to distinguish structurally different Caribbean (C-CTX), Indian (I-CTX) and Pacific Ocean (P-CTX) congeners. Even though they differ structurally, the common features that integrate these group of toxins is the long semi-rigid architecture that comprises trans/syn-fused ether ring with a molecular weight of 1023-1157 Da (Figure 3). Chemical structures of P-CTX [80,81,82,83,84,85,86] and C-CTX [87,88] are well-studied. They are heat-stable, highly oxygenated, lipid soluble cyclic polyethers. More than 20 analogues of P-CTX have been reported, with the main toxin groups being P-CTX-1, P-CTX-2 and P-CTX-3. Among these, P-CTX-1 is the most potent and thought to be responsible for the majority of neurological symptoms associated with ciguatera in the Pacific [81]. Ten analogues of C-CTX were identified by Pottier *et al.* [87]. C-CTX is the major analog group among the CTX toxin group. Four I-CTX toxin groups have been identified. I-CTX-1 & I-CTX-2 are the most common ones in comparison to I-CTX-3&I-CTX-4. The I-CTX-1 & I-CTX-2 have the same molecular weight (1140 Da) as C-CTX-1, with a closely related structure [89]. CTX poisoning occurs due to the ingestion of tropical reef fishes, which bioaccumulate the toxin from the dinoflagellate *Gambierdiscus* [90]. The CTX group causes cellular toxicities by elevating intracellular calcium concentration and by the binding and opening of non-selective, non-voltage activated ion channels, resulting in neurologic symptoms, such as hyperesthesia, paresthesia and dysesthesia which may appear from a few hours to two weeks after ingestion of a toxic specimen. Acute symptoms result in gastrointestinal and cardiovascular distress [91,92].

#### 1.2.4. Cyclic Imines

**Figure 4:**
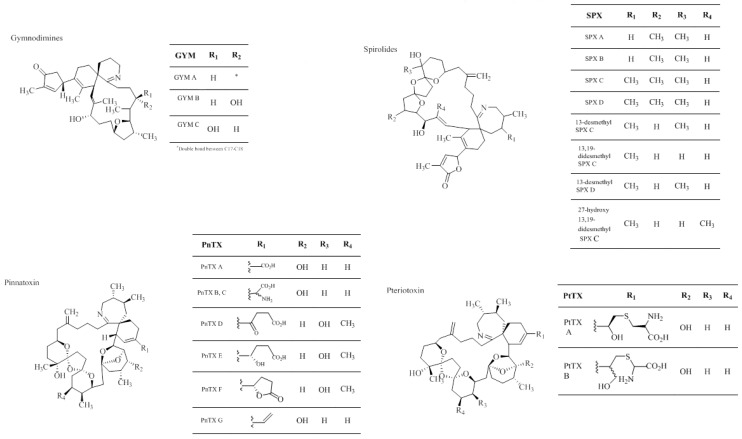
Gymnodimines (GYM) structure modified form Cembella and Krock 2008 [123]. Copyright 2008, Taylor & Francis. Spirolide (SPX) structure modified from Otero *et al.* 2010 [124]. Copyright 2010, Wiley. Pinnatoxin (PnTX) structure modified from Selwood *et al.* 2010, Copyright 2010, ACS Publications [114] and Rundberget *et al.* [112], 2011. Copyright 2011, Elsevier.

Cyclic imines (CI) are a group of toxins which include spirolides (SPXs), gymnodimines (GYMs), pinnatoxins (PnTXs) and pteriatoxins (PtTXs) produced by dinoflagellates. These toxins are macrocyclic compounds which share an imine functional group within their chemical structure (Figure 4) [93,94]. These cyclic imines are known as “fast-acting” toxins because they induce rapid death in the intraperitoneal mouse bioassay [95,96]. SPXs and GYMs are the largest group of CIs that are well-characterized. At present, 14 SPXs analogues have been isolated, whereby 13-desmethyl SPX-C is the most commonly one found in shellfish. In 1995, SPX was discovered in the Atlantic coast of Nova Scotia, Canada from mussels (*Mytilus edulis*) and scallops (*Placopecten magellanicus*) during the routine monitoring of lipophilic toxic compounds [97]. The spirolides toxin producing dinoflagellates, *Alexandrium ostenfeldii* and *A. peruvianum* were later described. Spirolides A–D are fast-acting toxins in mouse bioassay [98,99]. Spirolide E and F are biologically inactive with a keto-amine structure, which are the hydrolysis products of the Spirolides A–D [100,101,102]. This shows that imine group is important for the biological activity [100]. GYMs are produced by the dinoflagellate, *Karenia selliformis*. The structure of GYMs was first reported in 1995 by Seki and later confirmed by Stewart in 1997 by X-ray crystallographic analysis [103,104]. GYMs were first isolated from oysters (*Tiostrea chilensis*) coming from the South Island of New Zealand. The molecular mass of GYMs is 504.704 g/mol with a molecular weight of C_32_H_45_NO_4_. GYM-A has also been reported in Tunisia [105]. GYM-B and GYM-C were isolated from the coast of New Zealand as well. The structure of GYM-B is similar to GYM-A, but contains an exocyclic methylene at the C-17 position and an allylic hydroxyl group at the C-18 position, while GYM-C is an oxidized analog of GYM-A and was found to be isomeric with GYM-B at the C-18 position [106,107]. PnTX and ptTXs are closely related to the chemical structure of SPXs. The pinnatoxin also contains a number of analogues (PnTXs A-G). The first of these to be discovered was pinnatoxin A from the digestive gland extract of *Pinna attenuata* in China and Japan. Pinnatoxins B, C and D were isolated from viscera of the *Pinna muricata* [108,109,110]. Pinnatoxins E and F were found in the Pacific oysters (*Crassostrea gigas*) from Ranganau Harbour, Northland, New Zealand [111]. Pinnatoxin G was also isolated from the Norwegian blue mussel (*M. edulis*) [112]. Pinnatoxins E, F and G have also been isolated from Pacific oysters and razorfish (*Pinna bicolor*) from South Australia [113,114]. The organism responsible for pinnatoxins (the dinoflagellate, *Vulcanodinium rugosum*), was discovered only after the analysis of sediment samples from Rangaunu Harbour and the French Mediterranean coast. The species was also found in South Australia, China, Spain, Hawaii and Japan [115,116,117,118,119,120]. Pteriatoxins (A, B and C) were isolated in 2001 by Uemura and co-workers from *Pteria penguin*. Pteriatoxins A, B and C have the same polyether macrocycles as in pinnatoxin A. These CIs are fast-acting neurotoxins in laboratory animals which inhibit the nicotinic and muscarinic acetylcholine receptors (mAChR and nAChR, respectively) in the central and peripheral nervous system and at the neuromuscular junction causing death [121]. The lack of reports of acute intoxications caused by the consumption of contaminated sea products may be due to poor recognition of the adverse symptoms of a mild intoxication, such as tachycardia or gastric distress [122]. Moreover, the chronic effects are not yet fully understood, therefore this matter should be treated with caution and efforts should be made to disclose CIs’ acute and long term effects.

The presence of emerging toxins in temperate coastal waters has recently been reported and episodes of human poisoning usually follow [19,39,125,126]. Due to the lack of systematic data detecting these new toxins, a more comprehensive research strategy which better assesses the risk of public health is required. Some recent human intoxication episodes have alerted our attention. In October 2007, a Spanish man who consumed a trumpet shell (*Charonia lampas*) collected in the south of Portugal (Algarve) was severely intoxicated requiring hospital care. Analysis revealed the occurrence of TTX and 5,6,11-trideoxyTTX analogue in sublethal concentrations [19]. Ciguatoxin is a common toxin from Indo-Pacific and Caribbean waters that was first reported in Europe in 2003 in Greece. This toxin is produced by a dinoflagellate from the genus *Gambierdiscus* [127]. In July 2008, the intoxication of 11 crew members who ate carnivorous fish caught off the Madeira archipelago revealed the presence of CTX [39]. CIs are neurotoxic phycotoxins which were first reported in shellfish aquaculture in Nova Scotia, Canada in 1992 [97,100,102]. Their origin has been tracked to the dinoflagellates *Alexandrium ostenfeldii* and *A. peruvianum* [128]. Though they have acute neurotoxicity in mice, no human poisoning incidents have been reported to date [117,129]. CIs were reported along the North Atlantic and some groups of these biotoxins are confined to the Pacific Ocean [40,103,129,130,131,132,133]. PTX was first reported in Hawaii and Japan and their origin has been tracked to marine zoanthids, belonging to the genus *Palythoa*, and in dinoflagellates of the genus *Ostreopsis* [63,64]. Currently, blooms of *Ostreopsis* spp. have been reported in southern Europe indicating that the number of producers of this group of biotoxins is probably increasing from the Mediterranean Sea to the North Atlantic Ocean [134]. Also, since PTXs can form aerosols, several poisoning incidents have been reported among Mediterranean coastal populations as mild skin and respiratory disorders after exposure to high concentrations of *Ostreopsis* sp., luckily with no fatal outcomes [29].

These episodes suggest that there is an emergent phenomenon, indicating that marine toxins from tropical and subtropical ecosystems are most likely increasing their prevalence in temperate waters (Table 1). Multiple causes contribute to this phenomenon, such as the warming of coastal waters attributable to climate change and the increasing use of artificial waterways (*i.e.*, the Suez Canal) that allow for colonization and the establishment of exotic species in the Mediterranean Sea and the Atlantic Ocean [42,135,136,137]. Eutrophic areas of the Mediterranean Sea contribute to the formation of seed banks that provide favorable conditions for the establishment and migration of tropical organisms in more temperate areas of the North Atlantic.

**Table 1:** Detection of emergent poisoning incidents in the Mediterranean Sea and North Atlantic Ocean.

Toxin	Report location	Year	Vector/uptake route	No poisoning cases	Ref
**TTX**	Egypt/Israel	2005/2007/2008	*Lagocephalus sceleratus* (ingestion)	13	[136]
	Spain	2007	*Charonia lampas (ingestion)*	1	[19]
**PTX**	Italy	2005/2006	*Ostreopsis ovate* (aerosol)	228	[138]
	Spain	2010	*Ostreopsis sp. (aerosol)*	2	[139]
	France	2006–2009	*Ostreopsis sp. (aerosol/Dermic)*	47	[29]
**CTX**	Canary Islands	2004	*Seriola rivoliana* (ingestion)	5	[140]
	Madeira Island	2008	*Seriola sp.* (ingestion)	11	[39]
**CI**	-	-	-	-	-

## 2. Analytical Methods

Detection and quantification of the emergent toxins such as TTX, CTXs, CI and PTX have been based on different approaches (Table 2). Mouse bioassay (MBA) is the simplest method used for screening the total toxicity of the sample. In order to assess the toxicity, purified toxin samples or biological extracts are injected intraperitoneally and then animals are monitored for 24–48 h. The results are based on the biological response of mice and the toxicity of the sample is calculated in mouse units (MU). The relationship between time and lethal dose is used for estimation of the toxicity of the sample. This assay gives the total toxicity of a sample [141,142,143,144]. For screening and monitoring the toxins, many rapid, sensitive and specific assays (*i.e.*, cytotoxicity assay, immunological and receptor binding assays) have been developed. Cytotoxicity assay is used as the alternative method replacing the whole animal assays. This assay is based on the changes in the morphology of cells by the toxin. Cytotoxicity can be measured either through the lactate dehydrogenase (LDH) release assay or the MTT [3-(4,5-dimethylthiazol-2-yl)-2,5-diphenyltetrazolium] assay in the living cells at pg concentrations [143,145,146]. Immunoassays are antibody based assays used for the detection of toxins. These methods are very sensitive and allow for the detection of toxins in pg concentrations [147,148,149,150]. Receptor binding assays (RBA) are based on the principle of the affinity of the toxin to a specific binding site. The toxin can be measured by the binding reaction between a radiolabelled toxin and a non-radiolabelled toxin that binds specifically to the receptor. Mouse bioassay, receptor binding assay and immunological methods have been used for the analysis of these emergent toxins. It has been found that these methods are successfully used for the identification of toxins, but they fail to analyze the analogues or derivatives of these toxins. The immunological assay addressed for the analysis of the toxins involves the use of expensive antibodies and there are some ethical issues regarding the use of live animal for bioassays [54,151,152,153]. Analytical methods such as HPLC/MS, LC/MS/MS, GC-MS, LC-FLD and NMR have been developed, which are helpful for the identification of the structure and analogues of the toxin. The LC-FLD and GC-MS are not a good choice because those methods have difficulties in the quantification of toxins and its analogues, due to a large variation in the absorbance intensities. The non-volatile nature of some toxins should also be derived in the case of GC-MS analysis. LC-UV method does not provide proper selectivity for the toxins which lack chromophore structure. Therefore, LC-LC/MS is regarded as the best choice for the determination of emergent toxin and its analogues. Improvement in these methods and sample preparation decrease the limit of detection and quantification of the toxin [54,144,151,152,153].

## 3. Monitoring and Legislation Challenges

Safety management practices are required for shellfish due to the unpredictable nature of blooms [5,122]. Monitoring became the official strategy to control harvesting of shellfish areas throughout the world to prevent health and economical losses [122].

Monitoring is an essential, labour-intensive and costly activity. As a result of the Joint of FAO/IOC/WHO ad hoc Expert Consultation on biotoxins in bivalve mollusks held in 2004, guidelines for the organization of the marine biotoxin management plan (MBMP) were proposed. MBMP is based on several action plans that encompass an efficient sample strategy comprising periodicity and frequency, sample size and composition, and also, which analysis methods and managing action plans based on expert judgment of the results are the best and most effective [122].

Toxic phytoplankton species monitoring by itself is insufficient and strongly discouraged since it faces various inherent difficulties. It does not reflect the toxin content in shellfish species due to their intra and inter-specific differences nor their irregular distribution in the water column. Toxins can accumulate in bivalve species after the bloom has entered into a senescence state. Moreover, some toxin producing species may not be in the water suspension [154]. A good example is bacterial origin toxin TTX [154].

**Table 2:** Limit of detection/quantification of Emergent toxins (CTX, PTX, CI and TTX) by using different methods. (LOD—Limit of Detection, LOQ—Limit of Quantification, LD50—Lethal Dose 50%, SM—Shellfish Meat, mL—Milli liter, kg—Kilogram, µg—Microgram, ng—Nanogram, pg—Picogram, fg—Femtogram).

Assay	CTX	PTX	CI	TTX	Refs
**MBA**	LOD_P_-_CTX-1_ = 0.2 μg/kg SM LOD_C-CTX-1_ = 3.0 μg/kg	LD50 = 150–720 ng/µL	LOD_13–desMeC_ = 5.6 μg/kg SM LOD_GYM A_ = 77 μg/kg SM. *i.p.* LD_50PnTx E, F and G_ = 12.7–57 μg/kg SM	LOD_TTX_ = 0.2 µg	[114,129,142,143,144,155,156,157,158]
**Citotoxicity assay**					
Haemolysis assays	LOD = 50 µg/mL	LOD = 1.6 ng/kg SM 0.005 pg/µL–1 pg/mL	-	LOD = 5.0 µg/mL	[73,74,146,159,160]
Fluorimetric method	LOQ_C-CTX-1_ = 0.039 ng/g	-	-	-	-
Receptor-binding assays	LOQ_P-CTX-3Ceq_ = 15.5 fg/cul for algal samples 0.155 ng/g in fish samples	-	-	-	[161]
RBA with Neuroblastoma	LOQ_C-CTX-1_ = 0.039 ng/g	-	-	-	[162]
**Fluorescence polarization**					
Microsphere flow cytometry	-	-	LOQ_GYMA_ = 50–80 μg/kg LOQ_13–desMeC_ = 50–85 μg/kg SM LOQ_13,19–didesMeC_ = 40 μg/kg SM LOD_13–desMeC_ = 10 μg/kg SM	-	[163,164,165,166,167,168]
Chemiluminescence method	-	-	LOD_Spirolides_ = 50 μg/kg SM	-	-
Assays with MCF-7 cells	-	LOD = 0.5 ng/mL	-	-	[169]
Assays with neuroblastoma cells	-	LOD = 5 ng/mL	-	LOD = 3.2–160 ng/mL	[145,170,171]
**Immunoassays**					
Immunobead assay (MIA)	LOD_P-CTX-1_ = 32 ng/kg fish flesh	-	-	-	[147,172,173]
CIEIA	-	-	-	LOD = 10 ng/mL	[149]
ELISA	LOD = 0.28 ng/mL	LOD = 0.5 pg/mL	-	LOD = 5–50 ng/mL	[148,150,160,174,175]
Surface plasmon resonance (SPR)	-	-	-	100 µg/kg	[176]
**Chemical methods**					
HPLC-FLD/LC-FLD	LOD = 0.5–1.0 ng	LOD = 0.75 ng	-	LOD = 0.07 pmol–0.4 pmol	[73,177,178,179,180,181,182]
HPLC/MS	LOD_P-CTX-1_ = 4 ng/g	-	-	LOD = 2 ng/mL	[183,184]
HPLC-UV/LC-UV	-	LOD = 0.1–2 μg	LOD_GYM_ = 5 ng/mL	LOD = 10 ng/mL	[185,186]

MBMP on harvesting shellfish areas should be based on a combination of phytoplankton and shellfish to best assess the risk. It should be done periodically in order to timely detect the increase in toxin content in shellfish caused by the seasonal and spatial shifting in phytoplankton community. Samples should be representative of the area with adequate location and number of sampling sites that are reachable in all weather conditions. A good alternative to obtaining information about dissolved toxin content in water is through passive sampling techniques. Different resins can cover different toxins, reducing the cost and human effort and simplifying the analytical analysis [187]. This is due to the fact that the matrix effects are diminished, *i.e.*, phytoplankton matrices are less complex than shellfish meat [188]. However, there are some downsides to this methodology, as it does not cover all biotoxins. Firstly, it is not effective in the screening of toxins with bacterial origin and of benthic dinoflagellates. Moreover, toxins can be metabolized in shellfish and therefore the real risk for human consumers is not accurately measured [114,189,190]. In order to ensure representativeness, sampling must comply with important factors: samples should be gathered throughout the cultivation area, samples should represent all depths when a toxic event is in progress, shellfish must be gathered in all marketed sizes to address variability in toxin uptake, and samples should be in sufficient number in order to perform all the analyses needed. MBMPs ought to also gather atmospheric and hydrographic parametric information of the area along with an in-depth understanding of impending factors and their interactions. In order to see what the favorable conditions for the formation of a toxic phenomenon are, predictive modeling should be in place as well [122]. Finally, HABs can be also region specific, one good example of that is CTX [156]. Local and historical knowledge should be taken in consideration since it is often useful for targeting baseline studies prior to setting up a monitoring program, not only for CTX but for other potential emerging toxins.

Good practices are required for standardizing procedures. Emergent toxins pose a great challenge and answers are needed to address the issue of spreading to new temperate environments and trophic chains with unknown consequences. Standardization of the analytical procedures is urgently needed because contrary to other toxins like PSPs and DSPs, there is limited knowledge for emergent toxin routes, biochemical paths and standard reference materials. All of these contribute to the difficulties of monitoring and planning strategies for risk assessment.

The MBA is the most common method to assess phycotoxins in shellfish, although there are inherent difficulties with this method such as the ethics of using test animals and supply. The MBA also lacks specificity; identification of a toxin and its analogues or a mixture of toxins is not possible. Moreover, performing the test relies on toxin routes that are not extrapolated to humans. The purified extract of toxins is administrated via intraperitoneal injection, a different route from the common ones (oral, dermal or inhalation). Furthermore, extrapolating these results to humans relies on inter-specific errors. Given this, EFSA recommended the use of analytical methods such as the LC-MS. These procedures avoid the ethical issues. They are able to identify a toxin, in addition to its derivatives, in a mixture with a high degree of sensitivity, but as a downside analytical methods depend on reference standards for calibration. In terms of new emergent toxins, the need for standards is urgent. Currently only a few standards are available, meaning that the detection and quantification of these “new” toxins lack accuracy and are estimated according to their response factor. Data regarding acute reference dosage, median lethal dosage, legal limits in the European Union and standard availability on legislated toxins and on each group of emergent toxins will be described in more detail in Table 3.

**Table 3:** Summary of information of current and emergent toxins regarding: acute reference dosage (ARfD), median lethal dosage (LD_50_), legal limits in the European Union (EU) and standard availability, and comparison with regulated toxins PSP’s and OA group (DSP’s). (µg—microgram, eq—equivalents, g—gram, kg—kilogram, SM = Shellfish Meat, N.A.—not available).

Toxin group	Reference material	ARfD μg/kg bw	LD_50_ mice μg/kg bw	Legal limits in EU	Antidote	Refs
PSP	Yes ^(NRCC/Cifga)^	0.5 STX eq.	10	0.8 µg SXT eq/g SM	N.A.	[191]
OA	Yes ^(NRCC/Cifga)^	0.3	192	0.16 µg OA eq/g SM	N.A.	[192,193]
TTX	Lacking analogues ^(Cifga)^	2 *	9	2 μg of TTX eq/g SM *	N.A.	[194,195]
PTX	No certified material available	0.2	0.15–0.72	30 µg PLT eq/kg SM **	N.A.	[151,191]
CTX	No certified material available	N.A.	0.25	0.01 μg P-CTX-1 eq/kg fish ***	N.A.	[80,129,156]
CI	Lacking analogues ^(NRCC/Cifga)^	N.A.	5–8	400 μg CI/kg SM ****	N.A.	[152,196]

* Legislated limits for TTX are regarding the Japanese Government; ** EFSA recommends this value; *** EFSA recommends this value to cover all CTX-toxins; **** guidance value proposed by the EURLMB.

Analytical methods should comprise the whole animal but due to their high sensitivity, complex matrix interference may mask the results [41,197]. In order to overcome this problem, only the most affected organs are screened in some cases. This difficulty depends on the analyzed species, leading to another controversial issue: finding a species that can be used as an indicator. The commonly analyzed shellfish species are filter-feeders (mussels, scallops, cockles, oysters), and choosing a specific species, although it is advantageous in terms of cost reduction, is not effective since each species has different filtering and depuration rates. In addition to that, some studies showed that marine toxins risk assessment based on bivalves alone is redundant and misleading since some emergent toxins are not produced by phytoplankton [40,41]. These studies proved the possibility of bioaccumulation phenomena along the food-chain and reported new vectors for TTX and CIs from gastropods (*Monodonta lineata*, *Gibbula umbilicalis*, *Nucella lapillus*, *Aplysia depilans*, *Pattela intermedia*), to echinoderms (*Marthasterias glacialis* and *Paracentrotus lividus*) [40,41]. Likewise, CTXs have been detected from mollusks (ex: Turbinidae family) to top predator fish (ex: barracuda—Sphyraenidae family) occurring in the latter in higher concentrations suggesting that biotransformation and biomagnification could occur along the food-chain [122]. PTX poisoning incidents vary since all three exposure routes can occur (ingestion, inhalation and dermal) though there is a lack of proper reporting due to the difficulties inherent to identification/quantification owing to the absence of reference material [125,152]. This data shows that we are not analyzing the whole food-chain effectively, underestimating the risk for consumers.

As described in Table 3, certified material for emergent toxins is lacking. Regarding TTX, neither the toxin nor its analogs are regulated. There is only the Regulation (EC) No. 853/2004, which prevents the entry of products and derivatives belonging to the Tetraodontidae fish family in the EU [198]. Guidelines values and legislation only exist in Japan and Korea, where this family of fish is well appreciated. Chefs must be certified and the Japanese Ministry of Health and Welfare established the limit value of 2 μg of TTX equivalents/g SM [195].

In terms of PLT, there are no regulations globally. In 2009, EFSA recommended that PLT plus derivatives should not exceed 30 µg/kg SM [191]. There are also no regulations for CTX, only recommended values [156], and in some countries, fish with toxic provenance are prohibited from entering the market. That is true for the EU [198], Fiji, American Samoa, French Polynesia, Hawaii and Miami [199]. For CIs, though there is some toxicological information about SPX and GIM, there is still a lack of information on the other toxins of this group. Regarding certified material, there are only two groups that are characterized. Based on this, EFSA does not have enough information to establish the ARfD, since SPX is the only CI totally characterized so far [152]. Therefore, there is need to study the other groups in order to reach solid conclusions for creating safety measures.

Not much is known about the chronic effects of emergent toxins. Their appearance in temperate systems is quite recent and physicians may not be prepared to deal with these new symptoms of poisoning incidents. When a poisoning case appears, it is advised to query the patient, if possible, whether or not sea products had been consumed. If so, the patient will need to be admitted into hospital for gastric cleaning, ventilator and fluid support as there is no antidote available yet. Gastric content should be analyzed for confirmation of the poisoning agent.

Few epidemiologic reports exist which are crucial to understanding emergent toxin health risks. For that reason, it is also crucial to develop faster, more accurate and reliable methods of identification and qualification of these poisons to better help health professionals in their diagnosis and treatment.

## 4. Conclusions

The efficiency of risk assessment of marine toxins relies on the monitoring of HABs and risk evaluation of phycotoxins in fish and shellfish. Detailed epidemiological studies are needed to better evaluate safety levels and to promote regulations updates that will protect human health and reduce economic losses. An international effort must be made to share information, to optimize certified materials and to explore more expeditious and sensitive methods, such as chromatographic and molecular ones. All this becomes more relevant and urgent in the case of new emergent toxins like TTX, PTX, CI and CTX. In comparison to toxins, which are regulated (DSPs and PSPs), emergent toxins demonstrate higher lethality, with the exception of the CI, posing a potentially higher human health risk and thus requiring further research.
